# The Great Medical Slander Suit

**Published:** 1860-12

**Authors:** 


### THE GREAT MEDICAL SLANDER SUIT.

The jury empanelled in this noted case, which "dragged its slow length along" through some three or four week's trial, returned a verdict for the defendant.

Our readers are already in possession of the main facts. Mr. H. O. Stone was proved to have used language disparaging to the professional skill of Dr. Alexander Fisher, of this city, alleging malpractice and neglect on the part of the latter, in the obstetrical treatment of Mrs. Stone. At a period of some thirty-eight or forty days after the labor, *inversio uteri* was found to exist, and this proved the very *fons et origo mali* to all parties—the profession inclusive, the lawyers *ex*clusive. As we understand, on the one hand, that a new trial is applied for; and, on the other, that the whole pleadings, testimony, rulings, arguments, &c., are to be put in print, we deem it proper to forbear present comment. Suffice it to say, never was a hapless jury so pelted with abstractions, hailed upon by technicalities, so hopelessly befogged by professional literature, or so astounded by professsional wisdom, since the first doctor came into the first court. The dusty archives of medical history were exhumed, the corners of all the medical journals were ransacked; the dead lions were evoked, and the living came personally, or by written depositions of absolutely fabulous longitude, into the court. Legal acumen was exhausted in asking questions impossible of answer, or if answered having no conceivable relation to any case capable of being supposed, in this or any other sphere of human existence. Long may it be before we look upon the like again; and yet we could almost be tempted to listen once more, could we again have the pleasure of listening to such a model medical witness as Prof. White, of Buffalo, upon the stand proved himself to be. However one agreed or disagreed with the opinions he advanced, all coincide in awarding him high praise for his truly professional bearing; the clearness, precision, and force of his various replies, through two days and a half of pertinacious, if not always pertinent, questioning.

